# Insights into the recognition mechanism of shark-derived single-domain antibodies with high affinity and specificity targeting fluoroquinolones

**DOI:** 10.1007/s42995-024-00277-3

**Published:** 2025-02-13

**Authors:** Chang Liu, Guoqiang Li, Yuan Chen, Hong Lin, Limin Cao, Kaiqiang Wang, Xiudan Wang, Martin F. Flajnik, Jianxin Sui

**Affiliations:** 1 State Key Laboratory of Marine Food Processing and Safety Control, College of Food Science and Engineering, Ocean University of China, Qingdao, 266404 China; 2 School of Chemistry, Chemical Engineering and Biotechnology, Nanyang Technological University, Singapore, 637457 Singapore; 3 School of Medicine, University of Maryland, Baltimore, MD 21201 USA

**Keywords:** Shark-derived single-domain antibody, Fluoroquinolones, In silico, Alanine scanning mutagenesis, Binding mechanism

## Abstract

In this study, we investigated the molecular recognition mechanisms of shark-derived single-domain antibodies (ssdAbs) targeting fluoroquinolones using an integrated approach that combines in silico homologous modeling, molecular dynamics simulations, molecular docking, and alanine scanning mutagenesis. Three ssdAbs—2E6, 1N9, and 1O17—specific to enrofloxacin, norfloxacin, and ofloxacin, respectively, were selected based on previous work. Through AlphaFold2 and GalaxyWEB, the protein structures of these ssdAbs were predicted and optimized, followed by molecular dynamics simulations to emulate realistic protein behavior in a solvent environment. Molecular docking, alanine scanning mutagenesis, and subsequent verifications identified 30N and 93W of 2E6; 30N, 89R, 98Y, and 99D of 1N9; 100W and 101R of 1O17, all located within the complementarity determining region 3 loop, as critical for antigen binding. These residues primarily interact with their targets through hydrogen bonds, salt bridges, π–π stackings, and cation–π interactions. This study revealed, for the first time, the binding mechanism of ssdAbs to fluoroquinolones from a theoretical perspective, emphasizing the importance of aromatic and polar residues in recognizing characteristic epitopes, such as the carboxyl group at the C3 position and the 1-piperazinyl group at the C7 position. Our findings provide valuable insights for the rational design and enhancement of ssdAbs for detecting small molecule hazards in aquaculture.

**Supplementary Information:**

The online version contains supplementary material available at 10.1007/s42995-024-00277-3.

## Introduction

Fluoroquinolones (FQs) are among the most crucial family of antibiotics, characterized by a shared quinolone ring structure with various substitutions (Wang et al. 2022). In recent years, the efficacy of FQs has been thoroughly validated, leading to their widespread adoption, particularly in aquaculture. However, misuse and overuse of FQs in aquatic products pose significant risks to human health, including the development of antimicrobial resistance (Lin et al. 2023), liver damage (Pan et al. 2022), and renal failure (Ramesh et al. 2021). Consequently, there is a critical need for effective methods to detect FQs (Ding et al. 2024). Immunoassays, known for their rapidity and suitability for high-throughput screening, have been widely considered for this purpose (Cui et al. 2011; Wang et al. 2014). Despite their importance, traditional IgG antibodies used in these immunoassays have several limitations, including variability in sensitivity and specificity due to uncertain hapten rationality during the immune process (Wang et al. 2024; Xiao et al. 2024), difficulties in improving intrinsic properties after generation, and challenges in investigating the binding mechanisms between IgG antibodies and small molecules (Pan et al. 2022). Therefore, developing new antibody materials with high specificity and sensitivity, capable of directed transformation based on an understanding of binding mechanisms, is of significant importance for FQ detection in immunoassays (Wang et al. 2023).

In recent years, advances in genetic engineering have significantly promoted the development of engineered antibodies. Single-domain antibodies (sdAbs), in particular, have garnered attention for their potential in detecting small molecule hazards, owing to their advantages over traditional IgG antibodies (Hickey et al. 2022). First, the amino acid sequence of sdAbs can be precisely determined, allowing for rational maturation through analysis of the antigen-binding mechanism, thereby enhancing specificity and sensitivity at the molecular level (Huo et al. 2020). Second, sdAbs can be heterologously expressed with other antibody fragments or biomarkers to create hybrid functional elements in immunoassays. Finally, compared to traditional IgG antibodies, sdAbs are more animal-friendly, less time-consuming, and more cost-effective to produce (Windisch et al. 2024).

The increasing accuracy of protein predictive software has opened new possibilities for protein structure analysis (González-Fernández et al. 2024; Liu et al. 2023a). In silico approaches, often based on statistical models, such as Dead-End Elimination (DEE) and Monte Carlo algorithms, involve energy assessment, solvent treatment, and electrostatic interactions (Pal et al. 2021). Recently, machine learning and deep learning methods, such as trRossetta (Du et al. 2021) and AlphaFold2 (Jumper et al. 2021), have significantly improved the efficiency and accuracy of protein structure prediction. These methods leverage complex neural networks to combine evolutionary search with local structure suggestions (Vishwakarma et al. 2022). Additionally, advancements in Graphics Processing Unit (GPU) computing power and mathematical modeling have further propelled this field (Liu et al. 2022a). A vital advantage of these methods is their ability to build massive libraries, even without crystallized molecular structures, allowing for the simulation of antigen or antibody structures and their interactions (Tabasinezhad et al. 2019).

Despite the challenges associated with shark breeding and immunization, ssdAbs have shown promise as recognition materials for detecting small molecules. Shark serum contains high levels of penetrating salt ions and urea, making ssdAbs in IgNARs theoretically more stable than camel-derived single-domain antibodies (csdAbs) in extreme environments (English et al. 2020; Juma et al. 2021). In our previous studies, we successfully isolated ssdAbs targeting enrofloxacin (ENR), norfloxacin (NOR), and ofloxacin (OFL) from an immunized ssdAb phage display library, demonstrating high sensitivity and specificity (Liu et al. 2023b, 2024a,b). ENR-specific ssdAb 2E6, NOR-specific ssdAb 1N9, and OFL-specific ssdAb 1O17 (Amino acid sequences shown in Supplementary Fig. S1) were expressed through a soluble system, exhibited performance comparable to or better than traditional IgG antibodies and performed excellently in fish matrix tests. These ssdAbs met the detection requirements for ENR, NOR, and OFL in aquaculture, indicating their potential for practical application.

To further enhance the recognition performance of ssdAbs through directional evolution, we employed in silico approaches to predict the structures of these ssdAbs and their antigen-binding interactions. The predicted results were then experimentally verified to elucidate the actual binding mechanisms. This work not only clarifies the binding mechanism of ssdAbs to small molecule targets from a theoretical perspective but also lays the foundation for further structural–activity relationship studies and subsequent directional modifications, thereby enhancing the feasibility of ssdAbs in detecting small molecular hazards in aquaculture.

## Materials and methods

### Materials and instruments

Polymerase chain reaction (PCR) and Nanodrop One were purchased from Thermo Fisher Scientific Co., Ltd. (Shanghai, China). ENR, NOR, OFL, sodium dodecyl sulfate (SDS), and kanamycin were purchased from Aladdin Biochemistry Technology Co., Ltd. (Shanghai, China). The compounds ENR-PEI, NOR-PEI, OFL-PEI, ENR-OVA, NOR-OVA, and OFL-OVA—FQs conjugated to polyethylenimine (PEI) and ovalbumin (OVA) using the carbodiimide method, along with the mouse serum anti-V_NAR_, which broadly recognizes V_NAR_ ssdAbs, were produced by the Food Safety and Quality Control Laboratory at Ocean University of China (Shandong, China), as previously described (Liu et al. 2022b, 2024b). 3,3′,5,5′-*N*,*N*′-Tetramethylbenzidine (TMB), isopropyl β-D-1-thiogalactopyranoside (IPTG), glucose, *Ulp* protease kit, and HRP-goat anti-mouse serum were purchased from Solarbio Technology Co., Ltd. (Beijing, China). *E. coli* BL21 cells and Ni–NTA Sepharose were purchased from Sangon Biotech Co., Ltd. (Shanghai, China).

### Homologous modeling and optimization of protein models

The homologous modeling for ENR-specific ssdAb 2E6, NOR-specific ssdAb 1N9, and OFL-specific ssdAb 1O17 was performed using AlphaFold2 (Version 2.2.1). Five predicted structures for each ssdAb were generated (2E6AF1–2E6AF5; 1N9AF1–1N9AF5; 1O17AF1–1O17AF5) and further refined using the GalaxyWEB server (2E6AF1r–2E6AF5r; 1N9AF1r–1N9AF5r; 1O17AF1r–1O17AF5r). The structural rationality of these models before and after optimization was assessed using Ramachandran plots.

### Molecular dynamics simulation

Optimized protein structures were subjected to molecular dynamics simulation (MDS) using the Gromacs package (version 2020.6) to simulate realistic protein behavior in a solvent environment. The simulation workflow included topology generation, box and solvate definition, energy minimization, temperature and pressure equilibration, and production stages. The OPLS-AA/L force field was employed, and the simulation time was set to 1.0 ns. Root mean square deviation (RMSD) analysis was conducted to evaluate atomic motion during the simulation.

### Molecular docking

Molecular docking (MD) of the refined ssdAbs (2E6AF1rmds–2E6AF5rmds, 1N9AF1rmds -1N9AF5rmds, 1O17AF1rmds–1O17AF5rmds) with their respective ligands (ENR: PubChem CID: 71,188; NOR: PubChem CID: 4539; OFL: PubChem CID: 4583) was performed using Maestro software (Version 12.8.117). The MD process included protein preparation, ligand preparation, receptor grid generation, and docking stages. Each ligand was docked in 10 potential poses for further analysis.

### Alanine scanning mutagenesis, heterologous expression, and identification of mutant fusion proteins

Potential key amino acids (PKAAs) identified from MD analysis were mutated to alanine using site-directed mutagenesis. The original ssdAb genes (2E6, 1N9, 1O17) were mutated to generate variants, which were named accordingly (2E6-S1A, 2E6-N30A, 2E6-D91A, 2E6-W93A, 2E6-Y103A; 1N9-S1A, 1N9-N30A, 1N9-Y31A, 1N9-R89A, 1N9-Y98A, 1N9-D99A; 1O17-R90A, 1O17-E91A, 1O17-L96A, 1O17-S97A, 1O17-W100A, 1O17-R101A) (Wang et al. 2020). Mutant genes were cloned into the pET28a-SUMO plasmid for soluble expression in *E. coli* BL21 cells. The expression conditions were set according to those previously reported (Liu et al. 2024a, b). After induction with IPTG, cells were harvested, lysed, and the mutant proteins were purified using Ni–NTA columns. The purified proteins were verified by 12% SDS-PAGE.

### *Ulp* digestion and verification of mutant ssdAbs

To remove the SUMO tag protein, the *Ulp* protease digestion system was constructed as follows: 1 mg of mutant fusion protein, 20 μL of reaction buffer, 2 μL of *Ulp* Protease, and ddH_2_O to a final volume of 1 mL. The mixture was incubated at 4 °C for 16 h, followed by purification on Ni–NTA columns. The target mutant ssdAbs were verified by 12% SDS-PAGE.

### Validation of antigen-binding properties of mutant ssdAbs

The binding capabilities of 2E6, 1N9, and 1O17 mutant ssdAbs to the antigens ENR-PEI, NOR-PEI, and OFL-PEI were evaluated using indirect enzyme-linked immunosorbent assay (iELISA). Plates were coated with 100 μL of 30 μg/mL antigen solution and incubated at 4 °C for 16 h. After washing, 300 μL of mPBST (10 mmol/L PBS, pH 7.4, containing 5% (w/v) skimmed milk powder) was added for blocking at 37 °C for 2 h. In primary antibody stage, 100 μL of different diluted mutant ssdAbs was added and incubated at 37 °C for 1.5 h. After washing, 100 μL of mouse anti-V_NAR_ serum followed by 100 μL of HRP-goat anti-mouse serum was sequentially added, with each incubation at 37 °C for 1 h to complete the secondary antibody stage. After the final wash, 100 μL of TMB substrate solution was added for 10 min, and the reaction was terminated with 50 μL of 2.0 mol/L H_2_SO_4_. For blank control, the procedure was identical except that 100 μL of PBS was added in primary antibody stage.

Indirect competitive ELISA (icELISA) was employed to assess the sensitivity of 2E6, 1N9, and 1O17 mutant ssdAbs to ENR, NOR, and OFL, respectively. The procedure was similar to iELISA, except the coating antigens were replaced with 1 μg/mL of ENR-OVA, NOR-OVA, and OFL-OVA, respectively. In primary antibody stage, 100 μL of different diluted mutant ssdAbs was replaced with 100 μL of a mixture containing 50 μL of each mutant ssdAb and 50 μL of target solution at varying concentrations was pre-incubated at 37 °C for 30 min. The IC_50_ values for each mutant ssdAb with its respective target were calculated using four-parameter logistic equations as previously described (Han et al. 2020).

### Data analysis

Snapgene (Version 6.0.2, GSL Biotech Inc., Boston, USA) was used to analyze the integrity and homology of ssdAb genes. Homologous modeling of proteins was performed using AlphaFold2 (Version 2.2.1, DeepMind Technologies Ltd., Cambridgeshire, UK), followed by optimization with the GalaxyWEB server (Computational Biology Lab, Department of Chemistry, Seoul National University, Seoul, Korea). Gromacs (Version 2020.6, University of Groningen, Netherlands) was used for MDS, while MD was performed using Maestro software (Version 12.8.117, Schrödinger, Ltd., New York, USA). Data analysis and visualization were carried out using OriginLab (Version 8.1, OriginLab Inc., Massachusetts, USA) and Adobe Photoshop (Version 19.1.3, Adobe Systems Incorporated Inc., USA). Statistical analyses were performed with SPSS (Version 20.0, SPSS Inc., Chicago, USA), employing one-way ANOVA and Duncan’s test to assess significance, with *P* < 0.05 considered statistically significant.

## Results

### Analysis of protein models

**Fig. 1:**
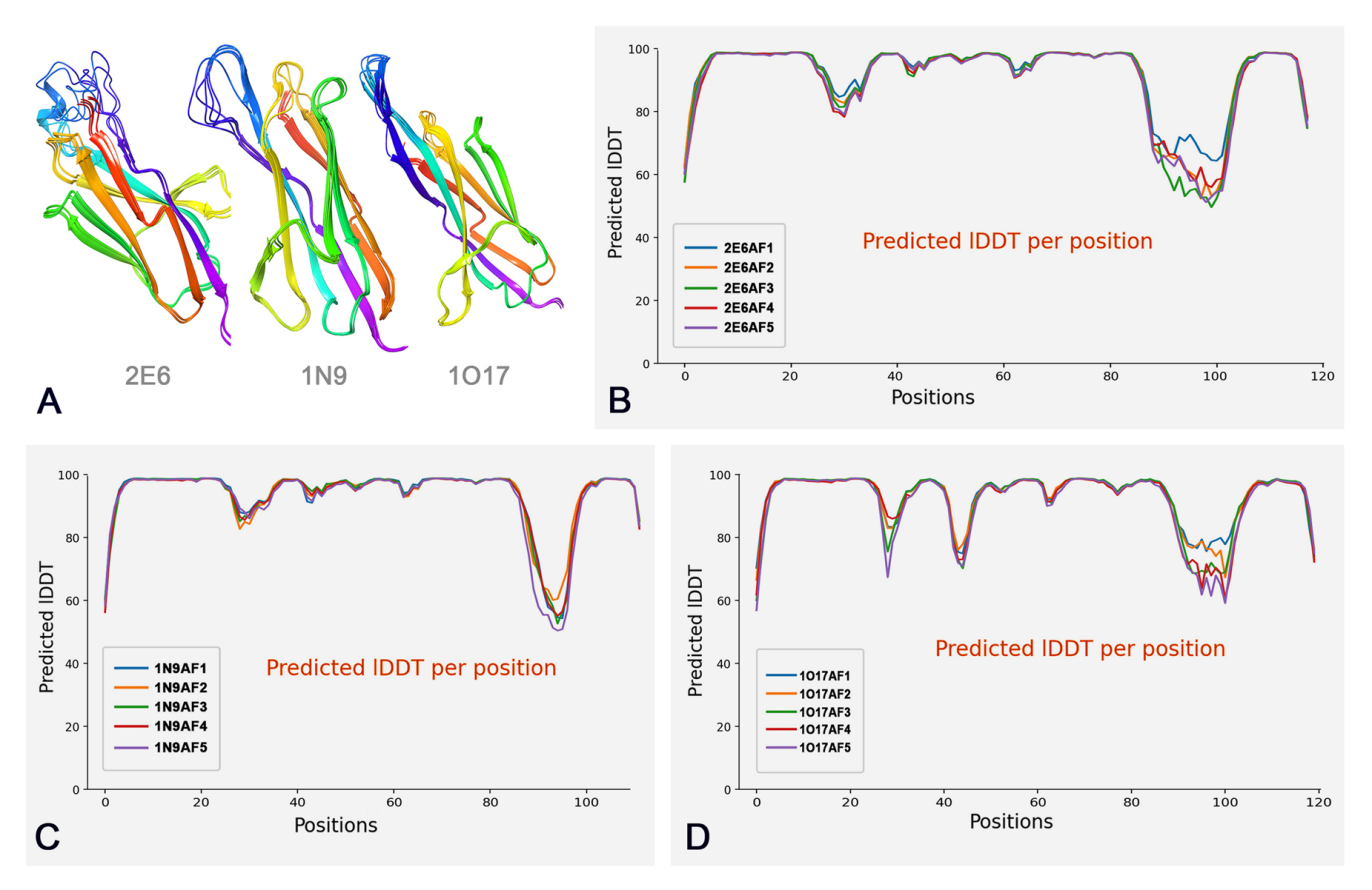
Schematic diagrams and plDDT plots of ssdAbs. **A** Represents the schematic diagrams of the 2E6, 1N9, and 1O17 protein models predicted by AlphaFold 2; **B**–**D** Represent the plDDT plots of the 2E6, 1N9, and 1O17 protein models, respectively

**Fig. 2:**
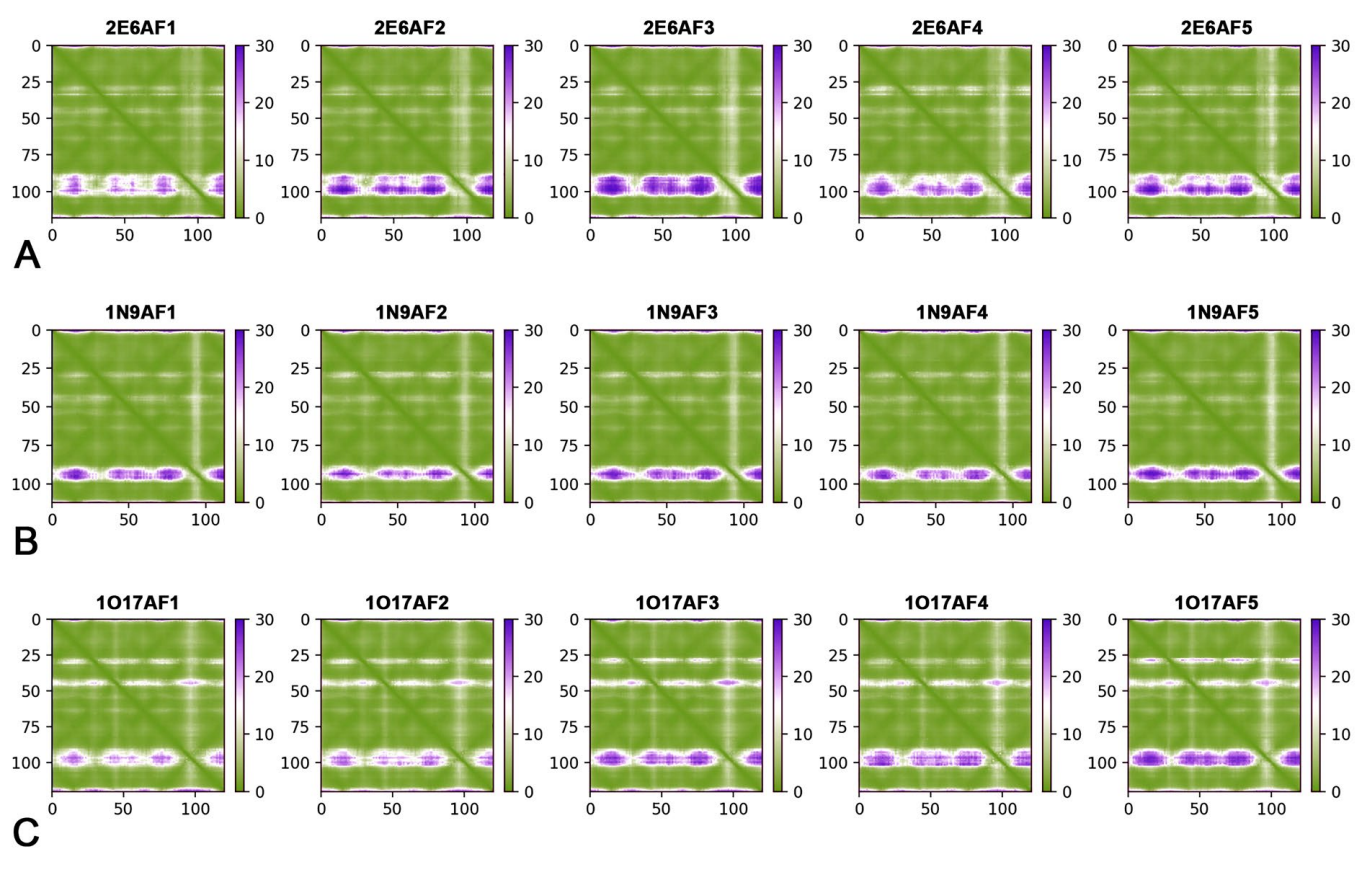
PAE plots of ssdAbs. **A**–**C** Represent PAE plots of the 2E6, 1N9, and 1O17 protein models, respectively

The AlphaFold Protein Structure Database, an open-access resource containing extensive high-precision protein structure prediction data, significantly expands the spatial structure coverage of known protein models (Tunyasuvunakool et al. 2021). Therefore, AlphaFold2 was employed to predict the protein structures of the 2E6, 1N9, and 1O17 ssdAbs. Upon inputting the amino acid sequences into AlphaFold2, five unrelaxed protein models were generated for each ssdAb: 2E6 (2E6AF1–2E6AF5), 1N9 (1N9AF1–1N9AF5), and 1O17 (1O17AF1–1O17AF5). These models were visualized as 3D schematic diagrams (Fig. 1A). The differences among the various prediction models for each ssdAb were primarily observed in the CDR3 loop, while other regions remained relatively conserved and overlapped in the diagrams. The plDDT plots (Fig. 1B–D) and PAE plots (Fig. 2) confirmed that most amino acid residues in each protein model aligned well with the predictive characteristics (Guo et al. 2022).

**Fig. 3:**
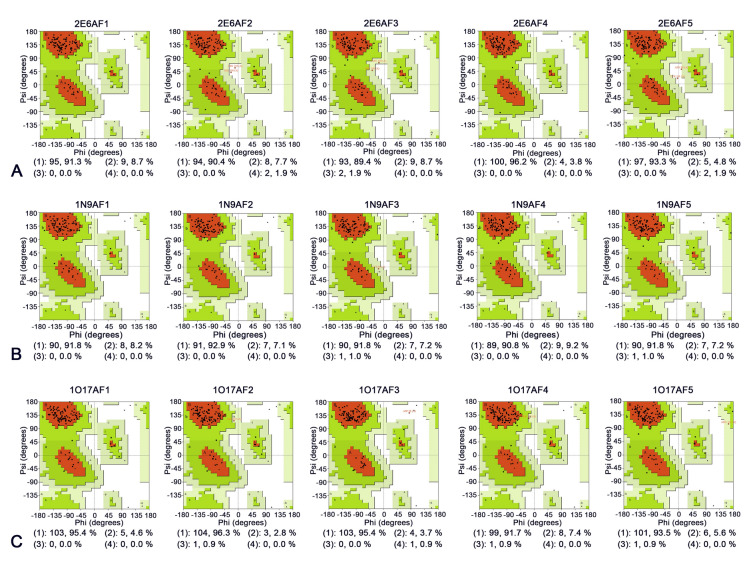
Ramachandran plots of ssdAbs before optimization. **A**–**C** Represent the Ramachandran plots of the 2E6, 1N9, and 1O17 protein models, respectively. (1) shows the number and percentage of residues in most favored regions [A, B, L]; (2) shows the number and percentage of residues in additional allowed regions [a, b, l, p]; (3) shows the number and percentage of residues in generously allowed regions [~ a, ~ b, ~ l, ~ p]; and (4) shows the number and percentage of residues in disallowed regions

**Fig. 4:**
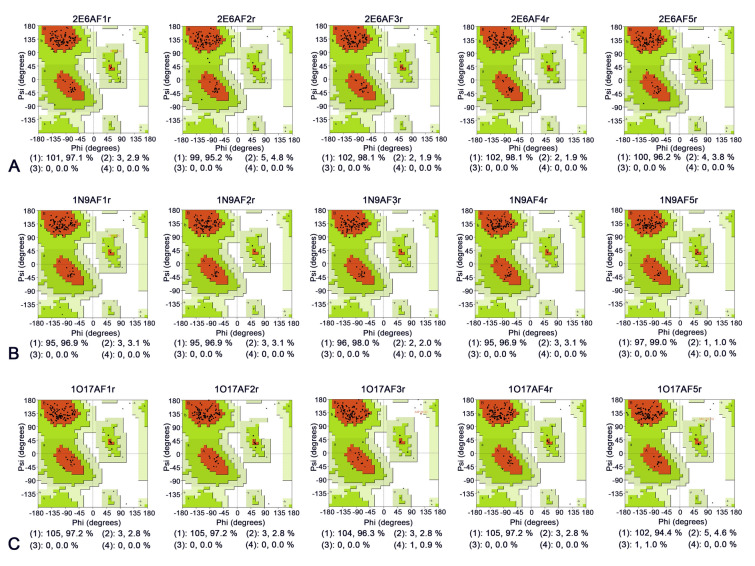
Ramachandran plots of ssdAbs after optimization. **A**–**C** Represent the Ramachandran plots of the 2E6, 1N9, and 1O17 protein models, respectively. (1) shows the number and percentage of residues in most favored regions [A, B, L]; (2) shows the number and percentage of residues in additional allowed regions [a, b, l, p]; (3) shows the number and percentage of residues in generously allowed regions [~ a, ~ b, ~ l, ~ p]; and (4) shows the number and percentage of residues in disallowed regions

Following optimization of the protein structures using the GalaxyWEB server, five optimized models were generated for each ssdAb: 2E6 (2E6AF1r–2E6AF5r), 1N9 (1N9AF1r–1N9AF5r), and 1O17 (1O17AF1r–1O17AF5r). The rationality of these optimized models was evaluated using Ramachandran Plots. As shown in Figs. 3 and 4, the “most favored regions” of the optimized protein models improved to varying degrees compared to the original models. This increase in “most favored regions” indicates amino acid residue distributions became more accurate. Among all protein models, 2E6AF3r showed the most significant improvement compared to 2E6AF3 in the “most favored regions”, as evidenced by the Ramachandran Plot results (from 89.4 to 98.1%).

### Analysis of MDS and MD

**Fig. 5:**
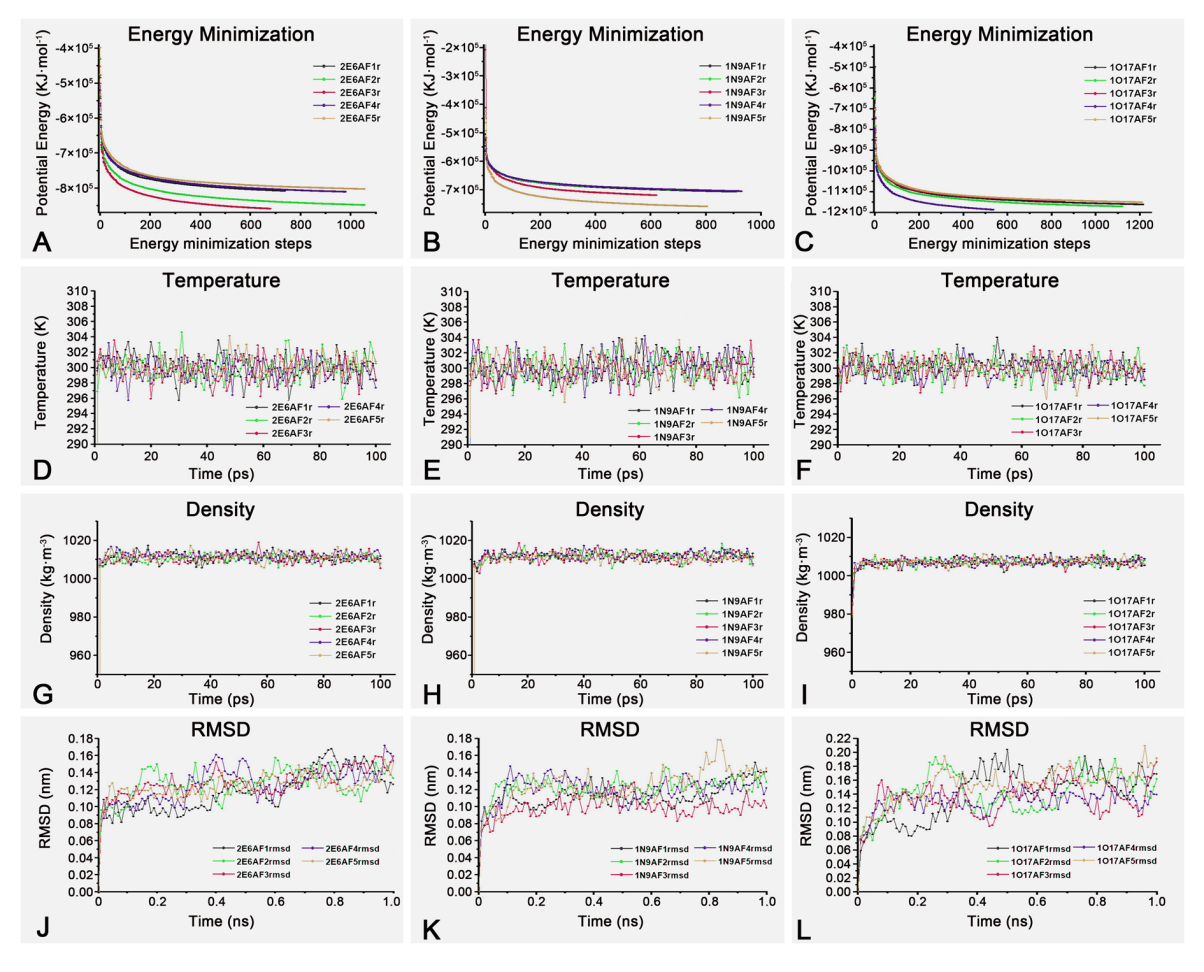
MDS results of ssdAbs. **A**–**C** Represent the energy minimization plots of the 2E6, 1N9, and 1O17 protein models before MDS, respectively; **D**–**F** Represent the temperature equalization plots of the 2E6, 1N9, and 1O17 protein models before MDS, respectively; **G**–**I** Represent the pressure equalization plots of the 2E6, 1N9, and 1O17 protein models before MDS, respectively; **J**–**L** Represent the RMSD plots of the 2E6, 1N9, and 1O17 protein models during MDS, respectively

To obtain more realistic protein structures in the presence of a solvent, MDS of all-atom molecules were performed in an SPC/E water solvent environment. Following the generation of the topology, energy minimization was applied to all protein models. As shown in Fig. 5A–C, the overall potential energy of 2E6AF1r–2E6AF5r, 1N9AF1r–1N9AF5r, and 1O17AF1r–1O17AF5r exhibited stable convergence during the energy minimization process. During the temperature and pressure equilibration stages (Fig. 5D–I), the solvent orientations of all models were corrected, and stability achieved as the temperature and pressure reached the set conditions.

After the MDS process, the resulting protein structures, denoted as 2E6AF1rmds–2E6AF5rmds, 1N9AF1rmds–1N9AF5rmds, and 1O17AF1rmds–1O17AF5rmds, were exported, and RMSD values were calculated throughout the simulation. The RMSD values increased rapidly within the first 10 ps and then fluctuated slightly between 10 and 1000 ps, representing the structural trajectory. As shown in Fig. 5J–L, the final RMSD values for 2E6AF1rmds–2E6AF5rmds, 1N9AF1rmds–1N9AF5rmds, and 1O17AF1rmds–1O17AF5rmds were less than 0.15 Å, 0.14 Å, and 0.18 Å, respectively, indicating all newly exported protein models were stabilized and suitable for subsequent MD process (Gorelov et al. 2024).

**Fig. 6:**
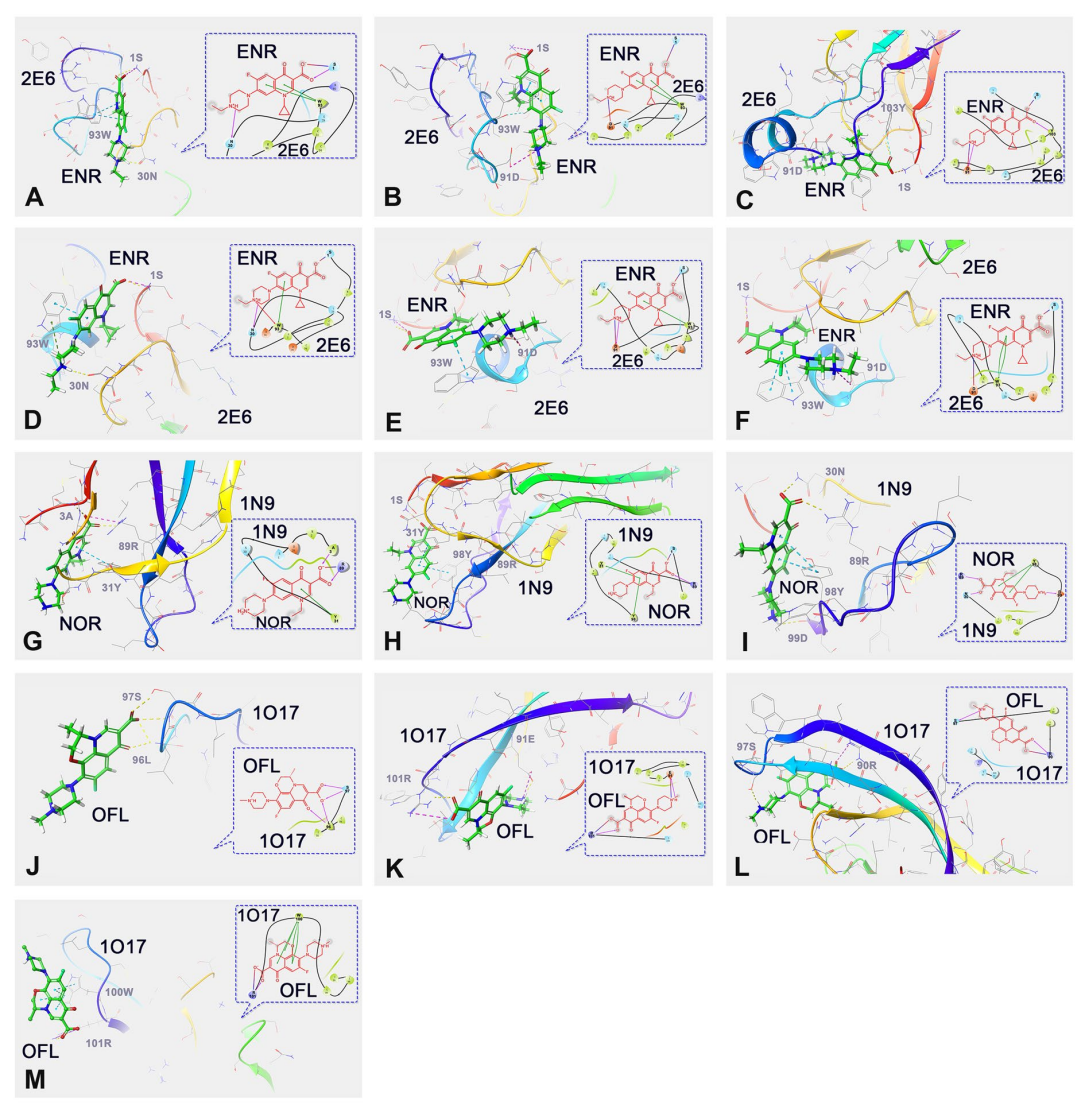
MD results for ssdAbs binding to target FQs. **A**–**F** represent the MD results of 2E6-Case 1 to 2E6-Case 6, respectively; **G**–**I** represent the MD results of 1N9-Case 1 to 1N9-Case 3, respectively; **J**–**M** represent the MD results of 1O17-Case 1 to 1O17-Case 4, respectively

Following the MDS process, the 2E6AF1rmds–2E6AF5rmds, 1N9AF1rmds–1N9AF5rmds, and 1O17AF1rmds–1O17AF5rmds protein models were subjected to MD analysis with their respective targets, ENR, NOR, and OFL, to identify potential key amino acids (PKAAs) and binding forces involved in antigen-binding interactions. After analyzing the docking results, six potential docking cases were identified for 2E6AF1rmds–2E6AF5rmds with ENR (Fig. 6A–F). The PKAAs involved in these interactions were 1S, 30N, 91D, 93W, and 103Y, which were associated with hydrogen bonds, salt bridges, π–π stackings, and cation–π interactions. Similarly, three potential docking cases were identified for 1N9AF1rmds–1N9AF5rmds with NOR (Fig. 6G–I), with PKAAs including 1S, 30N, 31Y, 89R, 98Y, 99D, involving hydrogen bonds, salt bridges, and π–π stackings. For 1O17AF1rmds–1O17AF5rmds with OFL, four potential docking cases were identified (Fig. 6J–M), with PKAAs including 90R, 91E, 96L, 97S, 100W, and 101R, also involving hydrogen bonds, salt bridges, and π–π stackings (Hutchinson et al. 2024; Zhao et al. 2024). All detailed PKAAs, along with the corresponding binding forces and distances, are summarized in Supplementary Table S1.

### Construction and identification of mutant ssdAbs

After identifying the PKAAs of the 2E6, 1N9, and 1O17 ssdAbs, alanine scanning mutagenesis was performed on all PKAAs to introduce site-specific mutations. The mutant ssdAbs were produced using a soluble heterologous expression system. The accuracy of the MD simulations, the identification of actual key amino acids (KAAs), and their interactions with targets were evaluated by comparing the antigen-binding abilities of these mutant ssdAbs with the original ssdAbs. Previous studies have demonstrated that the soluble expression system employed in this study can promote the correct folding of target proteins and the formation of atypical disulfide bonds, thereby enhancing the stability and solubility of the proteins (Shad et al. 2024). Therefore, the pET28a-SUMO plasmid was selected as the expression vector to achieve the soluble expression of the mutant ssdAb genes.

Transformants of 2E6 mutants (2E6-S1A, 2E6-N30A, 2E6-D91A, 2E6-W93A, 2E6-Y103A), 1N9 mutants (1N9-S1A, 1N9-N30A, 1N9-Y31A, 1N9-R89A, 1N9-Y98A, 1N9-D99A), and 1O17 mutants (1O17-R90A, 1O17-E91A, 1O17-L96A, 1O17-S97A, 1O17-W100A, 1O17-R101A) grew well and exhibited correct sequences, making them suitable for subsequent heterologous expression experiments. All mutant fusion proteins displayed target bands with the expected molecular weights (highlighted in Supplementary Fig. S2) in different target imidazole eluents, indicating successful soluble expression. The eluents containing the target fusion proteins were collected as summarized in Supplementary Table S2.

After appropriate concentration, the mutant fusion proteins were enzymatically digested using *Ulp* protease to separate the mutant ssdAbs from the SUMO tag proteins. As seen in the SDS-PAGE analysis, the bands corresponding to the mutant fusion proteins weakened or disappeared after enzymatic digestion (highlighted in red in Supplementary Fig. S3). New bands representing the mutant ssdAbs appeared in the binding buffer eluents (highlighted in green in Supplementary Fig. S3), with molecular weights matching the theoretical values for the 2E6, 1N9, and 1O17 mutant ssdAbs. These results indicate that the mutant ssdAbs were successfully separated from the fusion proteins and could be collected for further analysis. According to the protein concentration calculations, the yields of the 2E6, 1N9, and 1O17 mutant ssdAbs were all higher than those previously reported for co-expression with molecular chaperones (0.5–1 mg per 1 L culture medium) (Liu et al. 2014), demonstrating that our soluble expression system is effective for the expression of ssdAbs (Supplementary Table S2).

### Validation of MD predictions

**Fig. 7:**
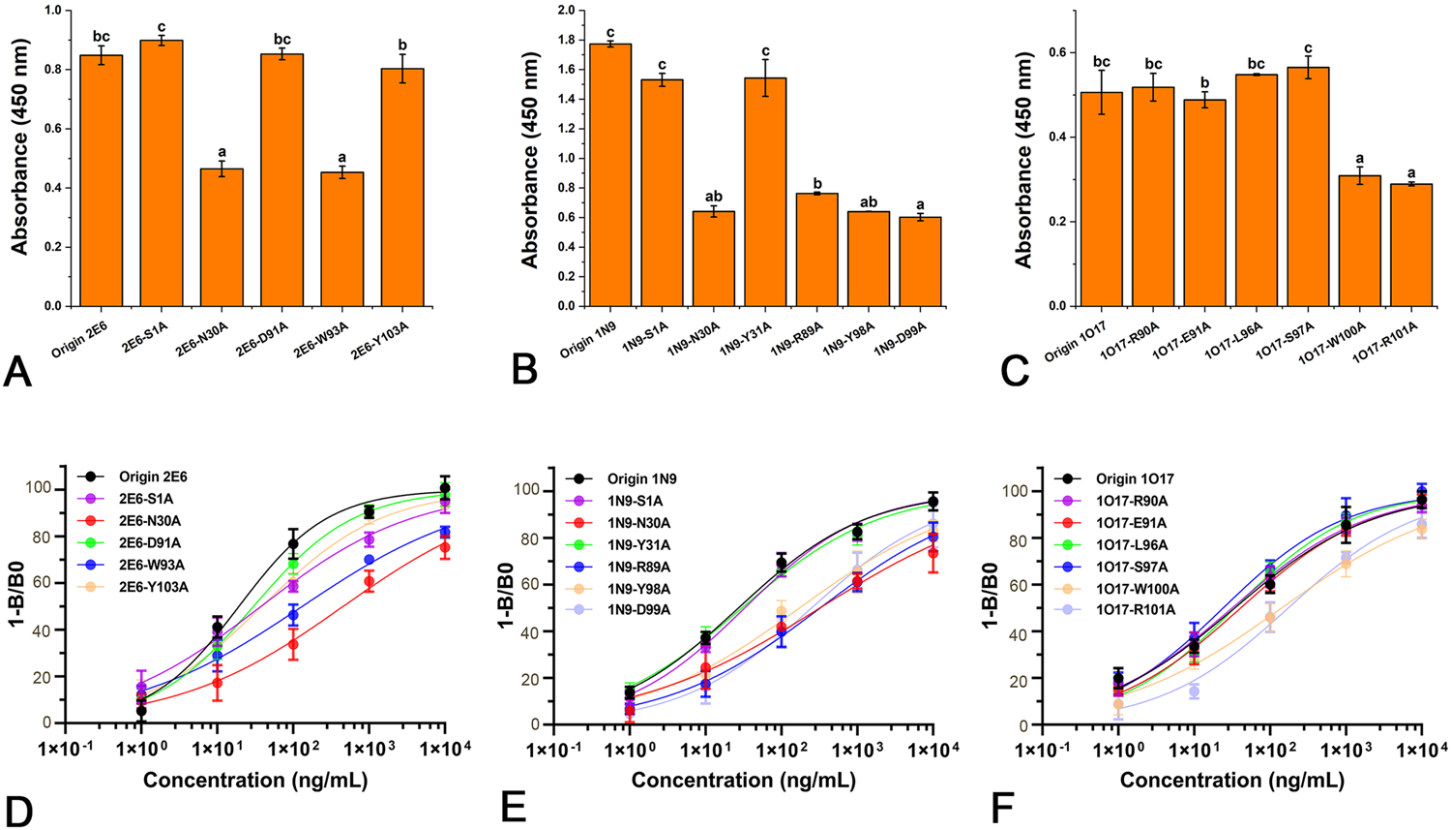
ELISA verification results of ssdAbs. **A**–**C** represent the iELISA results of the 2E6, 1N9, and 1O17 and their mutant ssdAbs binding to ENR, NOR, and OFL completed antigens, respectively; **D**–**F** represent the icELISA results of the 2E6, 1N9, and 1O17 mutant ssdAbs binding to ENR, NOR, and OFL. “a–c” indicate significant differences in OD450 values

The binding abilities of the 2E6, 1N9, and 1O17 ssdAbs and their mutant ssdAbs to their respective targets were carefully validated. As shown in Supplementary Figs. S4–S6, all 2E6, 1N9, and 1O17 mutant ssdAbs demonstrated binding abilities to the ENR, NOR, and OFL completed antigens, respectively. The OD_450_ values of the mutant ssdAbs at different dilutions showed significant variation, indicating that the binding of these mutant ssdAbs to the completed antigens was concentration-dependent. To further confirm changes in binding affinities of the mutant ssdAbs to FQs, additional verifications were conducted, and the results are presented in Fig. 7 and Supplementary Table S3. The IC_50_ values of the 2E6-N30A and 2E6-W93A mutant ssdAbs increased significantly by 6.8 and 23.6 times, respectively, compared to the original 2E6 ssdAb. Similarly, the IC_50_ values of the 1N9-N30A, 1N9-R89A, 1N9-Y98A, and 1N9-D99A mutant ssdAbs increased by 6.4–12.0 times compared to the original 1N9 ssdAb. In case of 1O17, the IC_50_ values of the 1O17-W100A and 1O17-R101A mutant ssdAbs increased by 4.2 and 5.1 times, respectively, relative to the original 1O17 ssdAb. The binding abilities of these mutant ssdAbs to the completed antigens were reduced to varying degrees across the board. In contrast, other mutant ssdAbs did not exhibit a significant impact on sensitivity to the targets, suggesting these particular PKAAs may not contribute to antigen binding. These experimental results strongly indicate that 30N and 93W of 2E6 ssdAb; 30N, 89R, 98Y, and 99D of 1N9 ssdAb; and 100W and 101R of 1O17 ssdAb are the actual KAAs in antigen binding.

## Discussion

In this study, we observed that KAAs responsible for binding interactions of ssdAbs with their targets were primarily located in the CDR3 loop. This finding is consistent with expectations, as the CDR3 region, characterized by its convex structure, provides an extensive surface area (approximately 600–800 Å) for antigen binding, contributing to the multifunctional flexibility of sdAbs (Stanfield et al. 2007). This feature is particularly prominent in ssdAbs, where the CDR3 loop can reach lengths of up to 19 amino acids, significantly longer than the 8–12 amino acids typically found in traditional IgG antibodies (Liu et al. 2022a). The longer CDR3 loop, combined with the unique gene cluster configuration and sequence diversification of ssdAbs, allows for more complex and varied paratope architectures, enabling effective antigen binding (Li et al. 2024). Although regions, such as CDR1, CDR2, and framework regions (FRs), are known to contribute significantly to antigen binding in csdAbs, our results indicate these regions are relatively conserved in ssdAbs and less involved in specific antigen interactions (He et al. 2024). The absence of the CDR2 region in ssdAbs is typically compensated by hypervariable regions HV2 and HV4. However, we did not observe significant contributions from these regions to antigen binding (Stanfield et al. 2007).

Our alanine scanning mutagenesis experiments demonstrated that mutating KAAs in 2E6, 1N9, and 1O17 ssdAbs negatively impacted their antigen-binding abilities. This is likely because these KAAs are directly involved in binding to their targets through various interactions, including hydrogen bonds, salt bridges, π–π stackings, and cation–π interactions. The replacement of even a single key residue with alanine can alter or disrupt the original paratope structure, thereby reducing the binding ability to the target molecules. This finding aligns with previous studies on small molecule target binding, where site-specific mutagenesis has been shown to have significant effects due to the fewer and more spatially flexible epitopes in small molecule antigens (Wang et al. 2020). Regarding the 2E6 ssdAb, the contributions of 30N and 93W to ENR recognition suggest that 2E6 ssdAb is most likely to bind ENR in the manner proposed as 2E6-Case 1 or 2E6-Case 4. In these scenarios, the 1S, 30N, and 93W amino acid residues were predicted to bind ENR through hydrogen bonds, salt bridges, π–π stackings, and cation–π interactions. However, the results showed no significant difference in the binding of ENR between the 2E6-S1A mutant ssdAb and the original 2E6 ssdAb, indicating 1S is not a KAA in the antigen-binding process. Consequently, 2E6-Case4, with a minor contribution from 1S, is more likely to represent the actual binding mode of 2E6 ssdAb to ENR. For the 1N9 ssdAb, the binding contributions of 30N, 89R, 98Y, and 99D perfectly matched 1N9-Case 3, while 100W and 101R of 1O17 ssdAb perfectly matched 1O17-Case 4, confirming the accuracy of our in silico predictions. The identification of 100W and 101R as KAAs in the 1O17 ssdAb is consistent with previous preliminary analyses of 1O17 ssdAb’s binding to its target OFL (Liu et al. 2024b). Although the conclusions reached in this study are consistent with previous research, the use of MDS and alanine scanning mutagenesis here allowed us to more precisely identify the KAAs involved in binding, providing a more accurate and robust understanding of the interaction mechanisms.

In this research, we identified aromatic and polar amino acids in the paratopes of ssdAbs as key factors in their ability to recognize different FQs. Aromatic residues in the KAAs of 2E6, 1N9, and 1O17 ssdAbs are capable of forming π–π stacking interactions with quinoline rings of FQs, which are characteristic epitopes of FQs. Additionally, polar residues in these KAAs facilitate the formation of hydrogen bonds and salt bridges with the active oxygen and nitrogen atoms of FQs, further enhancing specificity. This unique composition of sdAb paratopes, which differs from that of traditional IgG antibodies, may explain why ssdAbs exhibit comparable or even superior binding affinities (Abdo et al. 2024; Lee et al. 2024).

From the current analysis of KAAs, it is evident that the carboxyl group at the C3 position of ENR, NOR, and OFL plays a crucial role in binding to ssdAbs. The molecular conformation of the 1-piperazinyl group at the C7 position, particularly the presence of a 4-ethyl group in ENR, enables specific interactions with 30N and 93W of the 2E6 ssdAb, and 99D of the 1N9 ssdAb with the N4 of the 1-piperazinyl group of NOR. For OFL, the rearrangement of its quinolone ring into a benzoxazine allows the 100W residue of the 1O17 ssdAb to form multiple π–π stacking interactions (Liu et al. 2024c; Pan et al. 2022). These structural features may explain the specific recognition and binding of ENR, NOR, and OFL by the 2E6, 1N9, and 1O17 ssdAbs, respectively. Given the identified KAAs, future studies could consider introducing or substituting residues with similar properties in the CDR3 loop to further enhance the binding affinity of ssdAbs to their target FQs.

## Conclusions

This research elucidates the binding mechanisms of ssdAbs specific to FQs, revealing the critical roles of KAAs in the CDR3 loop. Through a combination of homologous modeling, MDS, MD, and alanine scanning mutagenesis, we demonstrated that the identified KAAs are essential for effective antigen binding, with their interactions primarily involving hydrogen bonds, salt bridges, π–π stackings, and cation–π interactions. This study highlights the importance of aromatic and polar residues in recognizing the characteristic epitopes of FQs, such as the carboxyl group at the C3 position and the 1-piperazinyl group at the C7 position. These insights advance our understanding of ssdAb-FQ interactions and lay the groundwork for further development and optimization of ssdAbs, enhancing their potential for application in food safety and environmental monitoring.

## Supplementary Information

Below is the link to the electronic supplementary material.

Supplementary file1 (PDF 1956 kb)

## Data Availability

Data will be made available on request.

## References

[CR1] Abdo AIK, Nordin F, Tye GJ (2024) Selection and evaluation of single domain antibody against p19 subunit of IL-23 by phage display for potential use as an autoinflammatory therapeutic. Int Immunopharmacol 137:112371 38852516 10.1016/j.intimp.2024.112371

[CR2] Cui J, Zhang K, Huang Q, Yu Y, Peng X (2011) An indirect competitive enzyme-linked immunosorbent assay for determination of norfloxacin in waters using a specific polyclonal antibody. Anal Chim Acta 688:84–89 21296209 10.1016/j.aca.2010.12.030

[CR3] Ding X, Ahmad W, Rong Y, Wu J, Ouyang Q, Chen Q (2024) A dual-mode fluorescence and colorimetric sensing platform for efficient detection of ofloxacin in aquatic products using iron alkoxide nanozyme. Food Chem 442:138417 38237297 10.1016/j.foodchem.2024.138417

[CR4] Du Z, Su H, Wang W, Ye L, Wei H, Peng Z, Anishchenko I, Baker D, Yang J (2021) The trRosetta server for fast and accurate protein structure prediction. Nat Protoc 16:5634–5651 34759384 10.1038/s41596-021-00628-9

[CR5] English H, Hong J, Ho M (2020) Ancient species offers contemporary therapeutics: an update on shark VNAR single domain antibody sequences, phage libraries and potential clinical applications. Antibody Ther 3:1–9 10.1093/abt/tbaa001 PMC7034638 32118195

[CR6] González-Fernández C, García-Álvarez MA, Cuesta A (2024) Identification and functional characterization of fish IL-17 receptors suggest important roles in the response to nodavirus infection. Mar Life Sci Technol 6:252–265 38827125 10.1007/s42995-024-00225-1 PMC11136934

[CR7] Gorelov S, Titov A, Tolicheva O, Konevega A, Shvetsov A (2024) Determination of hydrogen bonds in GROMACS: a new implementation to overcome memory limitation. J Chem Inf Model 64:6241–6246 39119674 10.1021/acs.jcim.3c02087

[CR8] Guo H, Perminov A, Bekele S, Kedziora G, Farajollahi S, Varaljay V, Hinkle K, Molinero V, Meister K, Hung C, Dennis P, Kelley-Loughnane N, Berry R (2022) AlphaFold2 models indicate that protein sequence determines both structure and dynamics. Sci Rep 12:10696 35739160 10.1038/s41598-022-14382-9 PMC9226352

[CR9] Han X, Lin H, Cao L, Chen X, Wang L, Zheng H, Zhang Z, Pavase TR, Wang S, Sun X, Sui J (2020) Hapten-branched polyethylenimine as a new antigen affinity ligand to purify antibodies with high efficiency and specificity. ACS Appl Mater Interfaces 12:58191–58200 33319977 10.1021/acsami.0c15586

[CR10] He L, Wu Q, Zhang Z, Chen L, Yu K, Li L, Jiang Q, Wang Y, Ni J, Wang C, Li Q, Zhai X, Zhao J, Liu Y, Fan R, Li Y (2024) Development of broad-spectrum nanobodies for the therapy and diagnosis of SARS-CoV-2 and its multiple variants. Mol Pharmaceutics 21:3866–3879 10.1021/acs.molpharmaceut.4c00165 38920116

[CR11] Hickey JW, Neumann EK, Radtke AJ, Camarillo JM, Beuschel RT, Albanese A, McDonough E, Hatler J, Wiblin AE, Fisher J, Croteau J, Small EC, Sood A, Caprioli RM, Angelo RM, Nolan GP, Chung K, Hewitt SM, Germain RN, Spraggins JM et al (2022) Spatial mapping of protein composition and tissue organization: a primer for multiplexed antibody-based imaging. Nat Methods 19:284–295 34811556 10.1038/s41592-021-01316-y PMC9264278

[CR12] Huo J, Bas AL, Ruza RR, Duyvesteyn HME, Mikolajek H, Malinauskas T, Tan TK, Rijal P, Dumoux M, Ward PN, Ren J, Zhou D, Harrison PJ, Weckener M, Clare DK, Vogirala VK, Radecke J, Moynié L, Zhao Y, Gilbert-Jaramillo J et al (2020) Neutralizing nanobodies bind SARS-CoV-2 spike RBD and block interaction with ACE2. Nat Struct Mol Biol 27:846–854 32661423 10.1038/s41594-020-0469-6

[CR13] Hutchinson M, Ruffolo JA, Haskins N, Iannotti M, Vozza G, Pham T, Mehzabeen N, Shandilya H, Rickert K, Croasdale-Wood R, Damschroder M, Fu Y, Dippel A, Gray JJ, Kaplan G (2024) Toward enhancement of antibody thermostability and affinity by computational design in the absence of antigen. Mabs 16:2362775 38899735 10.1080/19420862.2024.2362775 PMC11195458

[CR14] Juma SN, Gong X, Hu S, Lv Z, Shao J, Liu L, Chen G (2021) Shark new antigen receptor (IgNAR): Structure, characteristics and potential biomedical applications. Cells 10:1140 34066890 10.3390/cells10051140 PMC8151367

[CR15] Jumper J, Evans R, Pritzel A, Green T, Figurnov M, Ronneberger O, Tunyasuvunakool K, Bates R, Žídek A, Potapenko A, Bridgland A, Meyer C, Kohl SAA, Ballard AJ, Cowie A, Romera-Paredes B, Nikolov S, Jain R, Adler J, Back T et al (2021) Highly accurate protein structure prediction with alphafold. Nature 596:583–589 34265844 10.1038/s41586-021-03819-2 PMC8371605

[CR16] Lee NJ, Jung M, Yang HY, Shim H (2024) A single-domain antibody library based on a stability-engineered human VH3 scaffold. Sci Rep 14:17747 39085444 10.1038/s41598-024-68680-5 PMC11291719

[CR17] Li W, Chen M, Wang T, Feng X, Jiang X, Dong X, Zhang H, Tang X, Tian R, Zhang Y, Li Z (2024) Characterization and humanization of VNARs targeting human serum albumin from the whitespotted bamboo shark (*Chiloscyllium plagiosum*). Int J Bio Macromol 491:152039 10.1016/j.ijbiomac.2024.133082 38878923

[CR18] Lin T, Pan J, Gregory C, Wang Y, Tincher C, Rivera C, Lynch M, Long H, Zhang Y (2023) Contribution of the SOS response and the DNA repair systems to norfloxacin induced mutations in *E. coli*. Mar Life Sci Technol 5:538–550 38045542 10.1007/s42995-023-00185-y PMC10689325

[CR19] Liu JL, Zabetakis D, Brown JC, Anderson GP, Goldman ER (2014) Thermal stability and refolding capability of shark derived single domain antibodies. Mol Immunol 59:194–199 24667069 10.1016/j.molimm.2014.02.014

[CR20] Liu C, Lin H, Cao L, Wang K, Sui J (2022a) Research progress on unique paratope structure, antigen binding modes, and systematic mutagenesis strategies of single-domain antibodies. Front Immunol 13:1059771 36479130 10.3389/fimmu.2022.1059771 PMC9720397

[CR21] Liu C, Han X, Li G, Zhang T, Chen Y, Cao L, Lin H, Sui J (2022b) Branched polyethylenimine as a carrier for significantly improving the biopanning efficiency of phages specific to hapten. ACS Appl Polym Mater 4:5737–5745

[CR22] Liu X, Sigwart JD, Sun J (2023a) Phylogenomic analyses shed light on the relationships of chiton superfamilies and shell-eye evolution. Mar Life Sci Technol 5:525–537 38045544 10.1007/s42995-023-00207-9 PMC10689665

[CR23] Liu C, Lin H, Cao L, Wang K, Sui J (2023b) Characterization, specific recognition, and the performance in fish matrix of a shark-derived single-domain antibody against enrofloxacin. Talanta 265:124852 37385191 10.1016/j.talanta.2023.124852

[CR24] Liu C, Chen Y, Lin H, Cao L, Wang K, Wang X, Sui J (2024a) Shark-derived single-domain antibody as a new and effective recognition element for norfloxacin detection. J Food Compos Anal 133:106385

[CR25] Liu C, Chen Y, Lin H, Cao L, Wang K, Wang X, Sui J (2024b) The construction of ofloxacin detection in fish matrix based on a shark-derived single-domain antibody. Anal Chim Acta 1319:342986 39122284 10.1016/j.aca.2024.342986

[CR26] Liu Y, Luo Y, Li W, Xu X, Wang B, Xu X, Hussain D, Chen D (2024c) Current analytical strategies for the determination of quinolone residues in milk. Food Chem 430:137072 37549624 10.1016/j.foodchem.2023.137072

[CR27] Pal A, Mulumudy R, Mitra P (2021) Modularity-based parallel protein design algorithm with an implementation using shared memory programming. Proteins Struct Funct Bioinf 90:658–669 10.1002/prot.26263 34651333

[CR28] Pan Y, Yang H, Wen K, Ke Y, Shen J, Wang Z (2022) Current advances in immunoassays for quinolones in food and environmental samples. Trends Anal Chem 157:116726

[CR29] Ramesh M, Sujitha M, Anila PA, Ren Z, Poopal RK (2021) Responses of cirrhinus mrigala to second-generation fluoroquinolone (ciprofloxacin) toxicity: assessment of antioxidants, tissue morphology, and inorganic ions. Environ Toxicol 36:887–902 33382204 10.1002/tox.23091

[CR30] Shad M, Nazir A, Usman M, Akhtar MW, Sajjad M (2024) Investigating the effect of SUMO fusion on solubility and stability of amylase-catalytic domain from *Pyrococcus abyssi*. Int J Biol Macromol 266:131310 38569986 10.1016/j.ijbiomac.2024.131310

[CR31] Stanfield RL, Dooley H, Verdino P, Flajnik MF, Wilson IA (2007) Maturation of shark single-domain (IgNAR) antibodies: evidence for induced-fit binding. J Mol Biol 367:358–372 17258766 10.1016/j.jmb.2006.12.045

[CR32] Tabasinezhad M, Talebkhan Y, Wenzel W, Rahimi H, Omidinia E, Mahboudi F (2019) Trends in therapeutic antibody affinity maturation: from in-vitro towards next-generation sequencing approaches. Immunol Lett 212:106–113 31247224 10.1016/j.imlet.2019.06.009

[CR33] Tunyasuvunakool K, Adler J, Wu Z, Green T, Zielinski M, Žídek A, Bridgland A, Cowie A, Meyer C, Laydon A, Velankar S, Kleywegt GJ, Bateman A, Evans R, Pritzel A, Figurnov M, Ronneberger O, Bates R, Kohl SAA, Potapenko A et al (2021) Highly accurate protein structure prediction for the human proteome. Nature 596:590–596 34293799 10.1038/s41586-021-03828-1 PMC8387240

[CR34] Vishwakarma P, Vattekatte AM, Shinada N, Diharce J, Martins C, Cadet F, Gardebien F, Etchebest C, Nadaradjane AA, de Brevern AG (2022) VHH structural modelling approaches: a critical review. Int J Mol Sci 23:3721 35409081 10.3390/ijms23073721 PMC8998791

[CR35] Wang Z, Zhang H, Ni H, Zhang S, Shen J (2014) Development of a highly sensitive and specific immunoassay for enrofloxacin based on heterologous coating haptens. Anal Chim Acta 820:152–158 24745749 10.1016/j.aca.2014.02.043

[CR36] Wang X, Chen Q, Sun Z, Wang Y, Su B, Zhang C, Cao H, Liu X (2020) Nanobody affinity improvement: Directed evolution of the anti-ochratoxin A single domain antibody. Int J Biol Macromol 151:312–321 32084462 10.1016/j.ijbiomac.2020.02.180

[CR37] Wang M, Cetó X, Del Valle M (2022) A sensor array based on molecularly imprinted polymers and machine learning for the analysis of fluoroquinolone antibiotics. ACS Sens 7:3318–3325 36281963 10.1021/acssensors.2c01260 PMC9706806

[CR38] Wang C, Qin F, Tang S, Li X, Li T, Guo G, Gu C, Wang X, Chen D (2023) Construction of graphene quantum dots ratiometric fluorescent probe by intermolecular electron transfer effect for intelligent and real-time visual detection of ofloxacin and its L-isomer in daily drink. Food Chem 411:135514 36724609 10.1016/j.foodchem.2023.135514

[CR39] Wang B, Liu L, Zhang H, Wang Z, Chen K, Wu B, Hu L, Zhou X, Liu L (2024) A group-targeting biosensor for sensitive and rapid detection of quinolones in water samples. Anal Chim Acta 1301:342475 38553128 10.1016/j.aca.2024.342475

[CR40] Windisch R, Soliman S, Hoffmann A, Chen-Wichmann L, Danese A, Vosberg S, Bravo J, Lutz S, Kellner C, Fischer A, Gebhard C, Monte ER, Hartmann L, Schneider S, Beier F, Strobl CD, Weigert O, Peipp M, Schündeln M, Stricker SH et al (2024) Engineering an inducible leukemia-associated fusion protein enables large-scale ex vivo production of functional human phagocytes. Proc Natl Acad Sci USA 121:e2312499121 38857395 10.1073/pnas.2312499121 PMC11194515

[CR41] Xiao J, Qin L, Zhao D, Huang N, Xu W, Zhang L, Pan X, Han S, Ding M, Li L, Le T, Peng D (2024) Monospecific and ultrasensitive detection of ofloxacin: a computational chemistry-assisted hapten screening strategy and analysis of molecular recognition mechanism. J Hazard Mater 465:133221 38103295 10.1016/j.jhazmat.2023.133221

[CR42] Zhao J, Li P, Abd El-Aty AM, Xu L, Lei X, Gao S, Li J, Zhao Y, She Y, Jin F, Wang J, Hammock BD, Jin M (2024) A novel sustainable immunoassay for sensitive detection of atrazine based on the anti-idiotypic nanobody and recombinant full-length antibody. Chem Eng J 491:152039 38882000 10.1016/j.cej.2024.152039 PMC11173377

