# Supplementary material for: Insights into the recognition mechanism of shark-derived single-domain antibodies with high affinity and specificity targeting fluoroquinolones
Source: Mar Life Sci Technol. 2025 Feb 13;7(2):340–51. doi: 10.1007/s42995-024-00277-3 (PMC12102038; doi:10.1007/s42995-024-00277-3)
Supplement: Supplementary file 1 — Supplementary file1 (PDF 1956 kb) [file 42995_2024_277_MOESM1_ESM.pdf]

Supplementary File

Running Title: Binding mechanism of ssdAbs to FQs

Title: Insights into the recognition mechanism of shark-derived single-domain antibodies with high affinity and specificity targeting fluoroquinolones

Chang Liu[a, b], Guoqiang Li[a], Yuan Chen[a], Hong Lin[a], Limin Cao[a], Kaiqiang Wang[a], Xiudan Wang[a], Martin F. Flajnik[c], Jianxin Sui[a,*]

[a] State Key Laboratory of Marine Food Processing & Safety Control, College of Food Science and Engineering, Ocean University of China, Qingdao 266404, China

[b] School of Chemistry, Chemical Engineering and Biotechnology, Nanyang Technological University, Singapore 637457, Singapore

[c] School of Medicine, University of Maryland, Baltimore MD 21201, United States of America

[*] Corresponding author. E-mail: suijianxin@ouc.edu.cn. ORCID: 0000-0002-1556-0305.

1. Amino acid sequences of 2E6, 1N9, and 1O17 ssdAbs

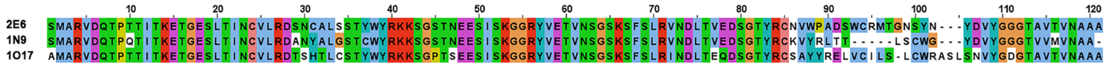

Fig. S1 The amino acid sequences of 2E6, 1N9, and 1O17 ssdAbs.

2. Detailed MD results of ssdAbs to targets

Table S1 MD results of 2E6, 1N9, and 1O17 ssdAbs with targets ENR, NOR, and OFL

Targets	Binding cases	PKAAs-binding forces	Binding distances (Å)
ENR	Case 1	1S-hydrogen bond;	2.26
		1S-salt bridge;	3.01
		30N-hydrogen bond	2.35
		93W-two π-π stackings	3.92; 4.37
	Case 2	1S-salt bridge	4.81
		91D-salt bridge	4.23
		93W-two π-π stackings	4.03; 4.22
	Case 3	1S-salt bridge	2.73
		91D-salt bridge	2.85
		91D-hydrogen bond	1.97
		103Y-hydrogen bond	2.18
	Case 4	1S-salt bridge	2.68
		30N-hydrogen bond	2.02
		93W-π-π stacking	4.08
		93W-cation-π interaction	5.72
	Case 5	1S-salt bridge	3.05
		91D-salt bridge	2.91
		91D-hydrogen bond	1.91
		93W-π-π stacking	4.03
	Case 6	1S-salt bridge	2.78
		91D-salt bridge	3.71
		93W-two π-π stackings	4.20; 4.31
NOR	Case 1	3A-hydrogen bond	1.98
		31Y-π-π stacking	5.03
		89R-salt bridge	4.08
		89R-hydrogen bond	2.00

	Case 2	1S-salt bridge	2.79
		31Y-π-π stacking	4.32
		89R-hydrogen bond	1.94
		98Y-π-π stacking	4.37
	Case 3	30N-hydrogen bond	1.71
		89R-hydrogen bond	1.93
		98Y-two π-π stackings	5.05; 5.09
		99D-hydrogen bond	2.15
OFL	Case 1	96L-hydrogen bond	2.14
		96L-hydrogen bond	2.46
		97S-hydrogen bond	1.93
		97S-hydrogen bond	2.65
	Case 2	91E-salt bridge	4.65
		91E-hydrogen bond	1.79
		101R-salt bridge	4.94
		101R-hydrogen bond	2.07
	Case 3	90R-hydrogen bond	2.01
		90R-salt bridge	4.42
		97S-hydrogen bond	2.03
	Case 4	100W-four π-π stackings	4.16; 4.08; 3.87; 3.94
		101R-hydrogen bond	1.70
		101R-salt bridge	4.23

3. SDS-PAGE identification results of mutant fusion proteins

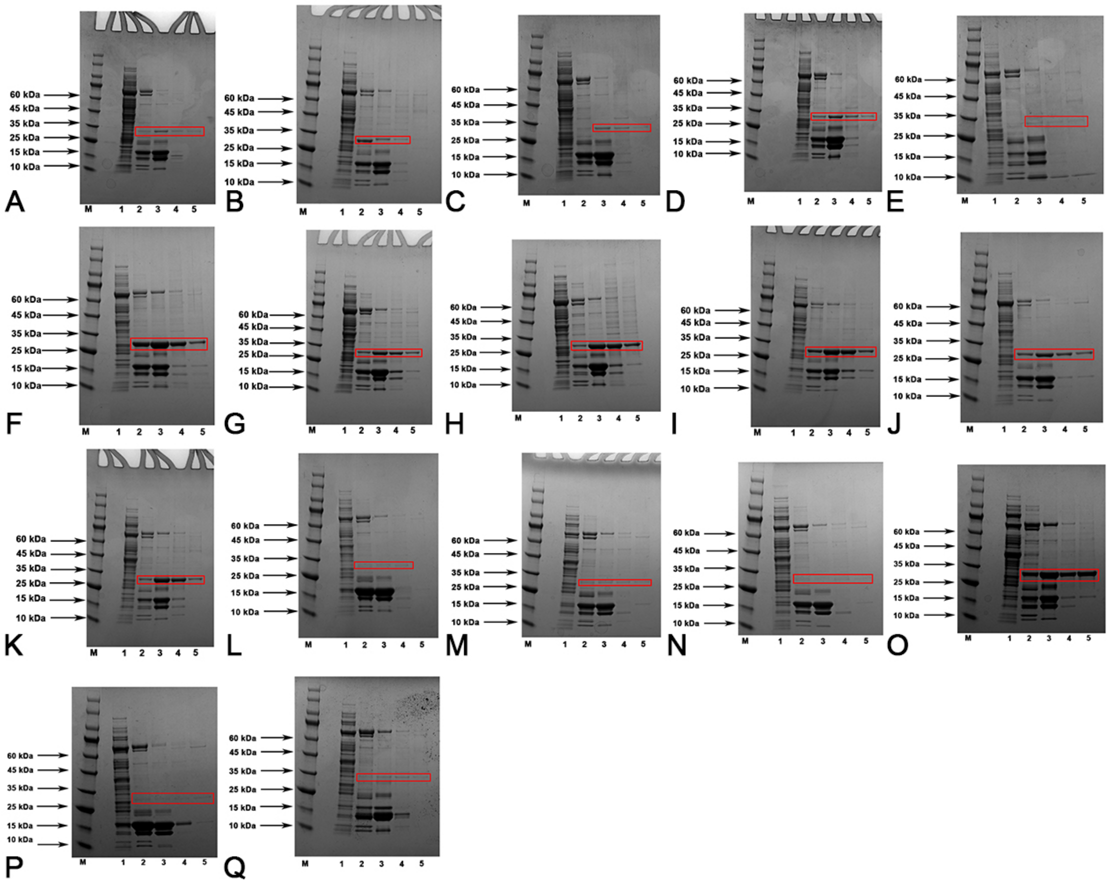

Fig. S2 A - E represent the SDS-PAGE identification results of different imidazole eluents for 2E6-S1A, 2E6-N30A, 2E6-D91A, 2E6-W93A, and 2E6-Y103A, respectively. F - K represent the SDS-PAGE identification results of different imidazole eluents for 1N9-S1A, 1N9-N30A, 1N9-Y31A, 1N9-R89A, 1N9-Y98A, and 1N9-D99A, respectively. L - Q represent the SDS-PAGE identification results of different imidazole eluents for 1O17-R90A, 1O17-E91A, 1O17-L96A, 1O17-S97A, 1O17-W100A, and 1O17-R101A, respectively. Band M represents the Protein Marker (180 kDa), and bands 1 - 5 represent the 20, 50, 100, 200, and 500 mM imidazole elusion components. The target fusion protein bands are highlighted in red.

4. Concentrations of collected imidazole eluents and yields of mutant fusion proteins

Table S2 Concentrations of collected imidazole eluents and yields of expression of 2E6, 1N9, and 1O17 mutant fusion proteins

Names of mutants	Concentrations of imidazole (mM)	Yields of expression (mg/L)
2E6-S1A	50, 100, 200, 500	2.50
2E6-N30A	50, 100, 200	2.50
2E6-D91A	100, 200, 500	1.25
2E6-W93A	50, 100, 200, 500	1.68
2E6-Y103A	100, 200, 500	1.64
1N9-S1A	50, 100, 200, 500	4.98
1N9-N30A	50, 100, 200, 500	5.12
1N9-Y31A	50, 100, 200, 500	4.82
1N9-R98A	50, 100, 200, 500	6.37
1N9-Y98A	50, 100, 200, 500	6.32
1N9-D99A	50, 100, 200, 500	4.40
1O17-R90A	50, 100, 200	6.88
1O17-E91A	50, 100, 200, 500	4.57
1O17-L96A	50, 100, 200, 500	4.38
1O17-S97A	50, 100, 200, 500	5.47
1O17-W100A	50, 100, 200, 500	6.63
1O17-R101A	50, 100, 200, 500	4.25

5. SDS-PAGE identification results of mutant ssdAbs

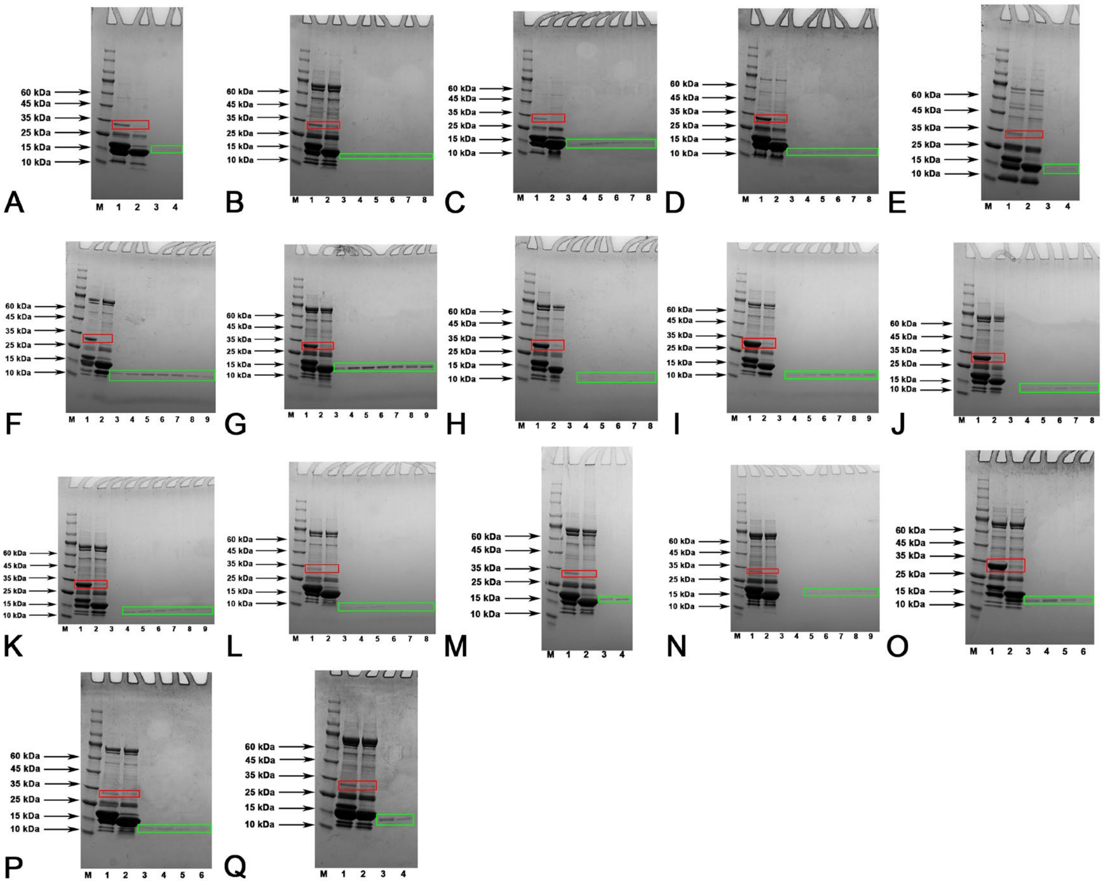

Fig. S3 A - E represent the SDS-PAGE identification of 2E6-S1A, 2E6-N30A, 2E6-D91A, 2E6-W93A, and 2E6-Y103A mutant fusion proteins before/after *Ulp* enzymatic digestion and the eluents from the binding buffer. F - K represent the SDS-PAGE identification of 1N9-S1A, 1N9-N30A, 1N9-Y31A, 1N9-R98A, 1N9-Y98A, and 1N9-D99A mutant fusion proteins before/after *Ulp* enzymatic digestion and the eluents from the binding buffer. L - Q represent the SDS-PAGE identification of 1O17-R90A, 1O17-E91A, 1O17-L96A, 1O17-S97A, 1O17-W100A, and 1O17-R101A mutant fusion proteins before/after *Ulp* enzymatic digestion and the eluents from the binding buffer. Band M represents the Protein Marker (180 kDa). Bands 1 - 2, highlighted in red, represent the fusion proteins before and after *Ulp* enzymatic digestion. Other bands, highlighted in green, represent the target mutant ssdAbs collected from the corresponding tubes.

6. ELISA verification of 2E6 and mutant ssdAbs

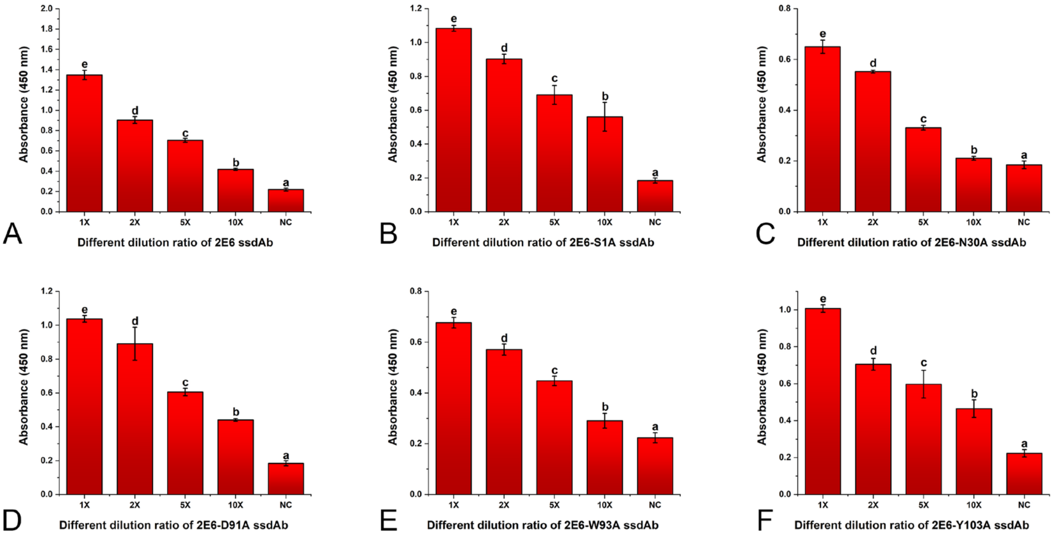

Fig. S4 ELISA verification results of 2E6 and mutant ssdAbs at different dilutions. "a - e" represent significant differences in OD_{450} values ($p < 0.05$).

7. ELISA verification of 1N9 and mutant ssdAbs

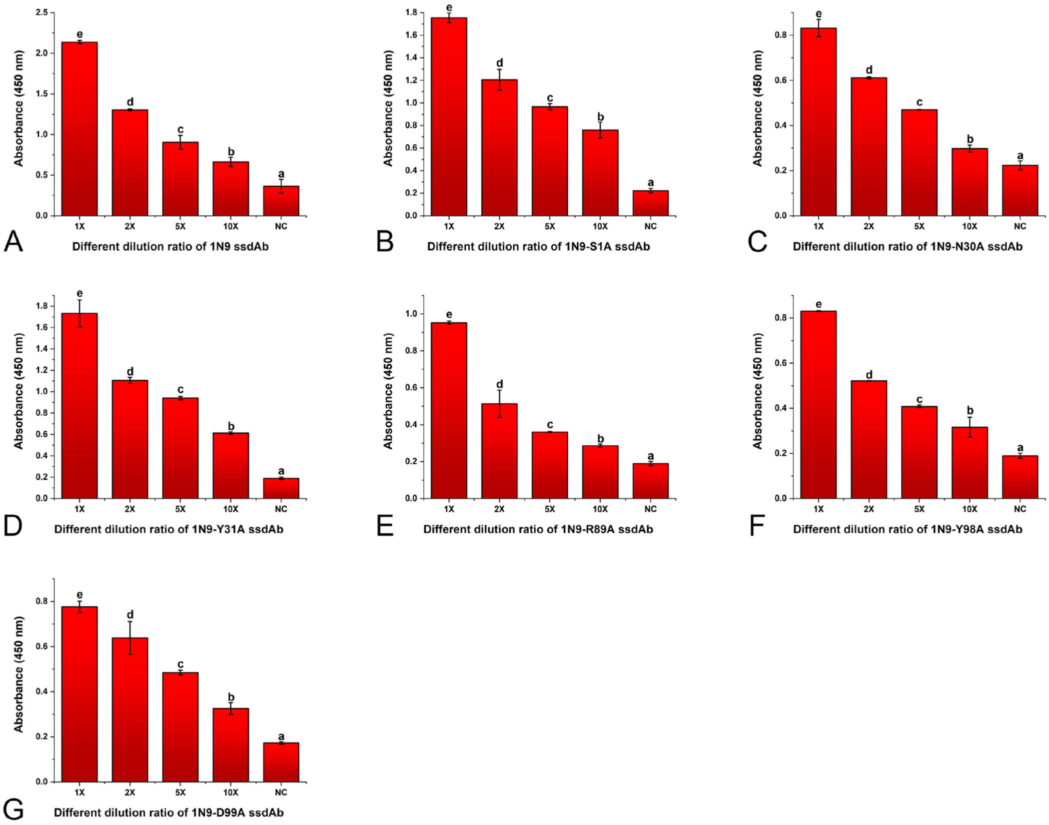

Fig. S5 ELISA verification results of 1N9 and mutant ssdAbs at different dilutions. "a - e" represent significant differences in OD_{450} values ($p < 0.05$).

8. ELISA verification of 1O17 and mutant ssdAbs

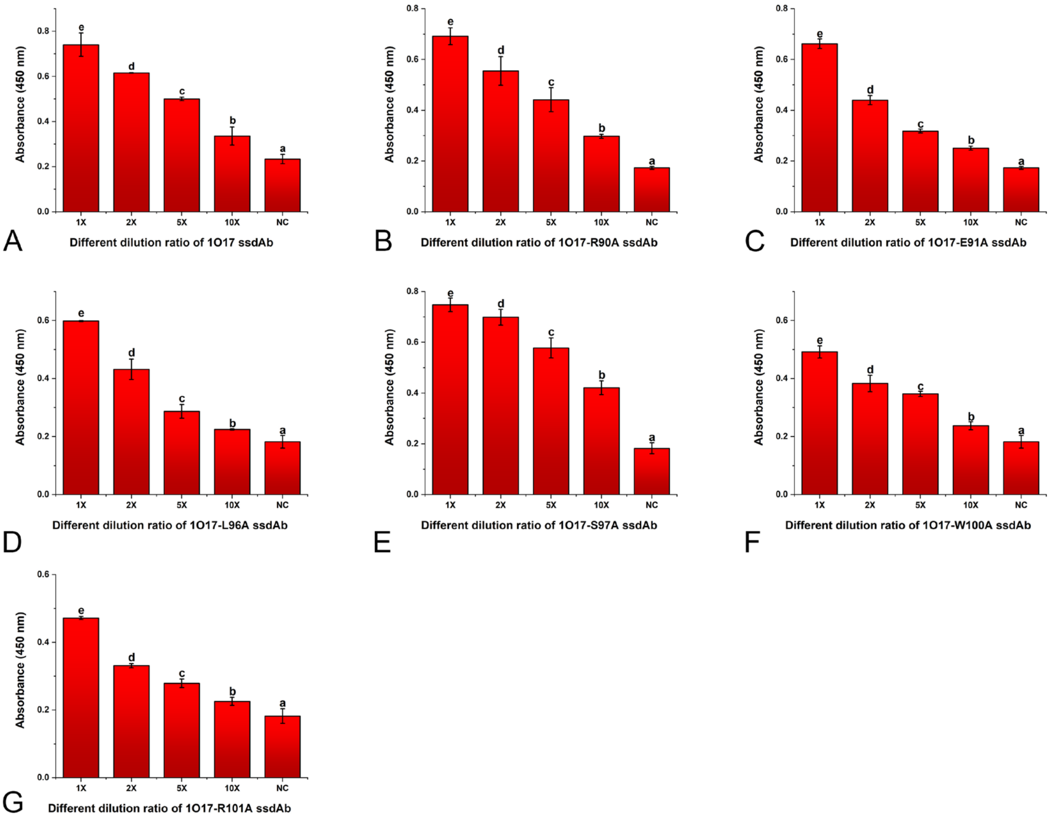

Fig. S6 ELISA verification result of 1O17 and mutant ssdAbs at different dilutions. "a - e" represent significant differences in OD_{450} values ($p < 0.05$).

9. IC_{50} results of ssdAbs to targets

Table S3 IC_{50} results of 2E6, 1N9, and 1O17 ssdAbs and their mutant ssdAbs to targets ENR, NOR, and OFL

Targets	Names of ssdAbs	IC_{50} values (ng/mL)
ENR	2E6	19.23
	2E6-S1A	37.52
	2E6-N30A	453.2
	2E6-D91A	29.93
	2E6-W93A	130.9
	2E6-Y103A	38.48
NOR	1N9	27.21
	1N9-S1A	32.89
	1N9-N30A	322.5
	1N9-Y31A	30.27
	1N9-R98A	325.4
	1N9-Y98A	173.7
	1N9-D99A	250.2
OFL	1O17	34.19
	1O17-R90A	31.75
	1O17-E91A	41.04
	1O17-L96A	34.22
	1O17-S97A	24.16
	1O17-W100A	142.6
	1O17-R101A	173.7
